# Supplementary material for: Thyroid Dysfunction and the Effect of Iodine-Deficient Parenteral Nutrition in Very Low Birth Weight Infants: A Nationwide Analysis of a Korean Neonatal Network Database
Source: Nutrients. 2022 Jul 25;14(15):3043. doi: 10.3390/nu14153043 (PMC9331788; doi:10.3390/nu14153043)
Supplement: Supplementary file 1 [file nutrients-14-03043-s001.zip › nutrients-1803797-supplementary.pdf]

Supplementary Materials

Table S1. Iodine content in manufactured parenteral and enteral formula based on the manufacturers' instructions.

Product	Iodine content (ug/100 mL)
Parenteral	
Primene 10% (Baxter, Ontario, Canada)	NA
TrophAmine (B.Braun Medical Inc, PA, US)	NA
SMOFlipid 20% (Fresenius Kabi Korea, Seoul, Korea)	NA
Omegaven 10% (Fresenius Kabi, Graz, Austria)	NA
Multitrace-4 Neonatal (American Regent Inc., NY, US)	NA
MVI Pediatric (Hospira Inc, IL, USA)	NA
Infuvite pediatric (Baxter,	NA
Enteral	
Absolute Premi (Maeil, Seoul, Korea)	8.4
Imperial Dream XO Premi (Namyang, Seoul, Korea)	7.0
Similac Sepcial care 20 (Abbott Nutrition, OH, US)	4.1
Enfamil Premature 20 Fe (MeadJohnson, IN, US)	16.9

Abbreviation: NA, not available.

Table S2. Multivariate stepwise backward regression analyses for the Outcome Assessment of Clinical Factors.

Outcome		Clinical factors	Odds Ratio	95% CI		*P*
				Lower	Upper	
Growth Retardation (< 5 percentile)	Weight	SGA	6.801	5.295	8.737	<0.001
		AED	2.945	1.583	5.479	0.001
		LT4	1.918	1.455	2.528	<0.001
		PN ≥ 4 weeks	1.705	1.363	2.132	<0.001
		BPD, mod to severe	1.497	1.209	1.853	<0.001
		Operation	1.439	1.144	1.810	0.002
		GA, per week decrease	1.134	1.076	1.196	<0.001
	Height	SGA	4.939	3.877	6.291	<0.001
		AED	4.349	2.323	8.142	<0.001
		BPD, mod to severe	1.491	1.210	1.836	<0.001
		PN ≥ 4 weeks	1.481	1.194	1.837	<0.001
		LT4	1.464	1.108	1.934	0.007
		Operation	1.290	1.025	1.622	0.030
		GA, per week decrease	1.086	1.032	1.144	0.002
	Head circumference	AED	9.308	4.477	19.349	<0.001
		SGA	4.595	3.442	6.135	<0.001
		PPHN	1.776	1.226	2.572	0.002
		PN ≥ 4 weeks	1.864	1.428	2.434	<0.001
		LT4	1.636	1.191	2.246	0.002
		Operation	1.630	1.262	2.106	<0.001
		BPD, mod to severe	1.622	1.267	2.075	<0.001
		RBC transfusion	1.535	1.041	2.263	0.030
		GA, per week decrease	1.087	1.020	1.157	0.010
Development	Cerebral palsy	AED	26.923	13.988	51.817	<0.001
		RBC transfusion	2.347	1.434	3.840	0.001
		Hypotension	1.691	1.260	2.270	<0.001
		PN ≥ 4 weeks	1.567	1.146	2.143	0.005
		Multi-gestation	1.563	1.194	2.046	0.001
		Drugs for PDA	0.654	0.497	0.860	0.002
		GA	10.625	4.027	28.030	<0.001
	Cognitive delay	AED	10.625	4.027	28.030	<0.001
		SGA	1.529	1.179	1.983	0.001
		PPHN	1.460	1.041	2.048	0.028

Operation	1.300	1.052	1.607	0.015
Sepsis	1.258	1.021	1.548	0.031
PN ≥ 4 weeks	1.195	0.976	1.465	0.085
GA	1.106	1.054	1.161	<0.001

P values were obtained by multivariate stepwise backward regression analyses. Abbreviations: SGA, small for gestational age; AED, antiepileptic drug; LT4, L-thyroxine; PN ≥ 4 weeks, Parenteral nutrition for ≥ 4 weeks; BPD mod to severe, Bronchopulmonary dysplasia with degree of moderate to severe; GA, Gestational age; PPHN, persistent pulmonary hypertension; RBC transfusion, red blood cell transfusion; PDA, patent ductus arterisosus.
